# The trend of marriage, childbearing, and divorce and its determinants of socio-economic factors on divorce in Yazd province 2016-2021: A cross-sectional study

**DOI:** 10.18502/ijrm.v21i8.14021

**Published:** 2023-09-20

**Authors:** Nastaran Tavakolian, Mohammad Hassan Lotfi, Moslem Taheri Soodejani, Farzan Madadizadeh, Foroozandeh Kalantari

**Affiliations:** ^1^Center for Healthcare Data Modeling, Departments of Biostatistics and Epidemiology, School of Public Health, Shahid Sadoughi University of Medical Sciences, Yazd, Iran.; ^2^Deputy for Health Affairs, Shahid Sadoughi University of Medical Sciences, Yazd, Iran.

**Keywords:** Family, Marriage, Childbearing, Divorce, Socioeconomic factors.

## Abstract

**Background:**

In recent decades, families and their stability as an important social institution have changed significantly.

**Objective:**

This study aimed to investigate the marriage trends, childbearing, and divorce changes in Yazd province from 2016 to 2021 to estimate the effect of socioeconomic factors on divorce.

**Materials and Methods:**

A cross-sectional study was done in 2 phases. In the first phase, an ecological (time trend) was conducted to investigate the 5 yr trend in the occurrence of marriage, childbearing, and divorce, as well as the factors affecting the occurrence of divorce in the second phase. For the second phase of the study, 600 participants were selected. 300 divorced and 300 married applicants were chosen between 2016 and 2021. A binary logistic regression model was used to find the related factors affecting the occurrence of divorce.

**Results:**

The results showed a declining marriage (p = 0.05) and childbearing trend (p = 0.84), as well as an increasing trend in divorces (p = 0.02) in Yazd. Logistic regression analysis showed that college education (OR = 0.22, CI: 0.116-0.430, p < 0.001) and being self-employed (OR = 0.48, CI: 0.255-0.934, p = 0.03) could reduce the odds of divorce. In addition, nonresidents (OR = 2.1, CI: 1.314-3.562, p < 0.001), with > 10-yr age differences (OR = 3.8, CI: 1.803-8.213, p < 0.001) or the woman being older than her husband (OR = 3.4, CI: 1.981-5.848, p < 0.001) could increase the odds of divorce.

**Conclusion:**

Our results confirmed that a combination of socioeconomic characteristics affects the stability of family institutions.

## 1. Introduction

The family is a significant social institution that has undergone changes in various societies throughout history (1). In recent times, families have experienced significant transformations, including increased female participation, reduced fertility, smaller family sizes, a shift toward nuclear families, a move away from traditional marriage norms, delayed marriage and childbearing, the emergence of new forms of relationships, rising divorce rates and separation, and an increase in woman-led families (2, 3).

One of the most critical dimensions of changing families is the spike in divorce rates. Globally, the divorce rate has increased over the past century (4, 5). This rising trend has been precipitating, especially since the 1960s, in many nations except a few countries such as Indonesia, Maldives, Taiwan, Malaysia, etc. (6). Despite this, a significant difference was observed between different societies in terms of the divorce rate. These differences should be viewed in light of each society's unique cultural and historical elements (7, 8).

Divorce rates in Iran have increased over the last decade. This rising trend can be seen across all provinces of Iran (9). In 1996, 8 cases of divorce out of 100 marriages were registered (10). The 2016's corresponding rate rose to 28 cases (11). The divorce rate in Iran rose from 94,039 in 2006 to 181,049 in 2016, an average increase of 0.61% per year (12). Marriage and divorce rates in the Yazd province in 2016 were 7.5 and 1.4, respectively, for every 1000 people (Provincial Civil Status Registry Organization, 2016). Yazd is one of the provinces that experiences an increase in the annual divorce rate over the past 10 yr (13). While in terms of the ranking of provinces, Yazd has the lowest divorce ratio compared to the province's total population (14). Due to long-held values, divorce rates in Iran are lower than other countries. However, considering the norms and standards of Iranian culture (where divorce was considered an abomination), an increase in the growth rate of divorce has been observed (15, 16).

Divorce has many effects and consequences on divorced men and women, their children, and society. An increased number of divorce cases and shorter marriages can also bring harm and social risks (9, 10). Various studies have shown that several factors can be the leading cause of divorce in a society. However, social and cultural factors (age difference, lack of dialogue culture, conflict of values due to media and social networks, heterogeneity of couples, etc.), economic factors and inflation, individual factors (level of education, etc.), and psychological factors (violence, etc.) are the 4 main aspects affecting the durability of marriage and family (15, 17, 18).

This study aimed to investigate the changes in marriage, childbearing, and divorce in Yazd province and determine the socioeconomic and demographic factors affecting the occurrence of divorce in this province using the available data.

## 2. Materials and Methods 

### Study design

**Figure 1:**
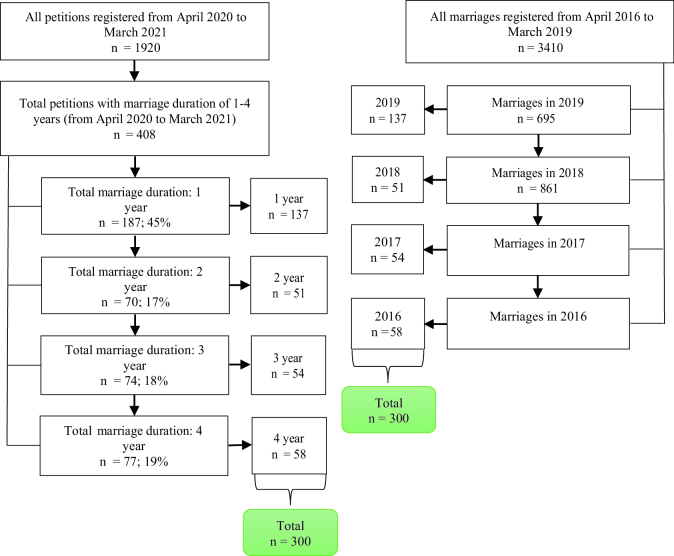
How to randomly selected paricipants for second phase of the study.

This cross-sectional study was conducted in 2 phases. In the first phase, to examine the trends, all marriages, children born, and divorces registered from April 2016 to March 2021 were used. The required sample size for second phase was 600 people that were selected randomly. A total of 300 individuals who initiated divorce proceedings from April 2020 to March 2021 were selected, in which participants had a marriage duration between 1-4 yr. Also, another 300 individuals were chosen who have been married between April 2016 and March 2019 and have not divorced. These groups were matched by duration of marriage (Figure 1).

The participants for the second phase were selected by simple random sampling method. In the end, social and economic factors, including job, education, and demographic characteristics such as gender, being resident (native and non-native residents of Yazd province), and the age difference between the couples according to year and gender; in both groups were compared.

### Data gathering 

In this study, all marriages, children born, and divorces registered in the civil registry of Yazd province, General Department of Justice of Yazd province and notary public No. 93 from April 2016 to March 2021 were considered. These data included age, education, job, being resident, duration of the marriage, total fertility rate, and the number of children at the time of divorce.

To determine the suitable sample size for the second phase of the study, the formula shown below has been used. This formula considers the average exposure to the risk factor in both groups, indicated by $\bar{P}$ , along with the size of divorced group in comparison to married group, represented by K. ( α=%5 , β=0.1 , K = 1). 


$$n=\frac{k+1}{k}\times \frac{(z_{1-\frac{\alpha }{2}}+z_{1-\beta })2}{({lnOR)}^{2}\bar{P}\left(1-\left.\bar{P}\right)\right.}$$

### Ethical considerations

In this study, the names of the participants were not considered and were approved by the Ethical Committee at the Shahid Sadoughi University of Medical Sciences, Yazd, Iran (Code: IR.SSU.SPH.REC.1401.064).

### Statistical analysis 

For described variables, frequency, percentage of frequency, mean, and standard deviation were used. The Cochran-Armitage linear trend test was also performed to examine changes in marriage, childbearing, and divorce trends. Also, binary logistic regression was used to investigate the predictive effects of social, economic, and demographic variables on divorce. All the analyses were performed by the Statistical Package for the Social Sciences, version 24.0, SPSS Inc. Chicago, Illinois, USA (SPSS) software (version 24) at a significance level of 5%.

## 3. Result

The average age of people at the time of marriage was 26.9 yr, which was 25 ± 6.05 yr for women and 28.9 ± 6.56 yr for men. Also, the average age of people when filing for divorce was 34 ± 8.9 yr, which was 32.7 ± 8.75, and 38.1 ± 11.6 yr for women and men, respectively. In 2016, most of the children born belonged to the age group of mothers aged between 25 and 29, while in 2021, the highest number of children born belonged to the group of mothers aged between 30 and 34.

Most people at the time of marriage had a bachelor's degree and were self-employed or had non-government jobs, while the most frequent ones among the divorce petitioners had a diploma and were housewives (Table I). The average duration of marriage at the time of the divorce petition was 11 ± 8.6 yr. While the shortest time reported was 3 days, and the longest time of living together was 57 yr. 66.7% of people had at least one child when filing for divorce.

**Figure 2:**
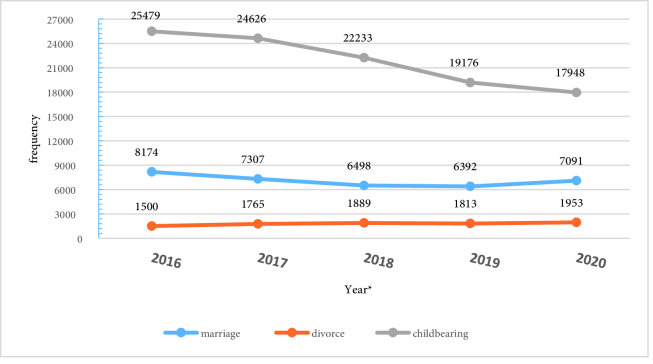
Frequency of marriage, childbearing and divorce in Yazd province by year with age range from 13-94 yr for marriages and divorces. The data reported for each year is from April of the same year to March of the following year.

According to the report of the General Directorate of Civil Registration of the Province, 35,462 marriages, 109,462 children were born, and 8920 divorce petitions were registered from April 2016 to March 2021 (Figure 2). In these 5 yr, the number of marriages and the number of childbearing have decreased by 13.2% and 29.5%, respectively. In addition, a 30.2% increase in the number of divorce petitions has also occurred. The Cochran-Armitage linear trend test showed that the trend of changes was significant only in the occurrence of divorce (p = 0.02), and the trend of changes in the occurrence of marriage (p = 0.05) and childbearing (p = 0.84) was not significant.

**Figure 3:**
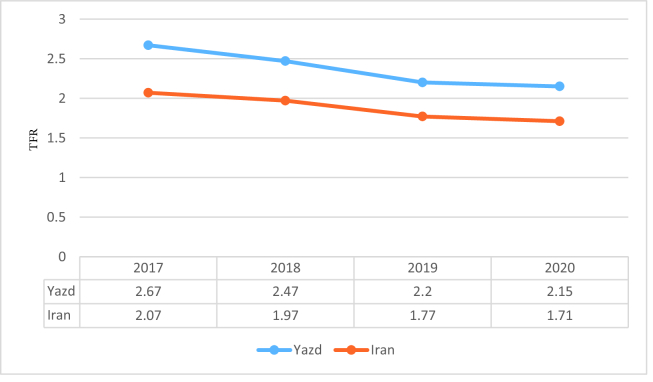
Total fertility rate of Yazd province and Iran from 2017-2020. TFR: Total fertility rate, *The data reported for each year is from April of the same year to March of the following year.

The current total fertility rate of Yazd province is 1.9-2.15 births per woman, close to the replacement level fertility (i.e., 2.1 births per woman). The total fertility rate declined from a maximum of 2.67 children per woman in 2017 to 2.15 children per woman in 2020; the TFR decreased by more than 24% over 4 yr (Figure 3).

Table II shows the demographic and socioeconomic characteristics of groups that were selected for second phase of the study. In both groups, most of the people were self-employed or had a nongovernment job, had a university education, and were residents of Yazd province. Most couples in both groups had an age difference of fewer than 5 yr, and men were older than women. All these factors except gender were significantly associated with the occurrence of divorce.

The crude and adjusted odds ratio of socioeconomic and demographic characteristics on the odds of divorce based on binary logistic regression is shown in table III. Regarding employment status, the results showed that the odds of divorce in people who had self-employed jobs decreased by 52% compared to unemployed people. Also, people who were housewives have 1.5 times higher odds of divorce, and among employees, the odds of divorce decreased by 47% compared to the unemployed, but these relationships were not statistically significant.

Compared to people with less than a diploma, people with a diploma 38% and university education 78% had less chance to experience divorce. However, the difference in the odds of divorce between people with a diploma and those without education was not statistically significant. Being a nonresident increased the odds of divorce by 2.1 times compared to resident people. In couples with an age difference of more than 10 yr, compared to those with an age difference of fewer than 5 yr, in terms of a divorce, the odds of divorce increased 3.8 times. Also, a woman being older than a man can increase the odds of divorce up to 3.4 times.

The study presented odds ratios based on socioeconomic and demographic variables for women and men separately in table IV. Employed women had a lower odd of divorce compared to those who did not have a job, and women with a university education had lower odds of divorce than those with less than a diploma. However, being a non-native resident with an age difference of > 5-10 yr or > 10 yr increased the odds of divorce for women, although these associations were not statistically significant.

The study found that men who were homemakers had significantly lower odds of divorce than men who did not have a job. Additionally, having a university education and being a non-native resident were associated with lower odds of divorce. However, having an age difference of more than 10 yr with one's spouse increased the odds of divorce significantly for men, unlike what was observed in women.

**Table 1:** Socioeconomic characteristics of married people and divorce applicants from April 2016 to March 2021 in Yazd province


**Variables**		**Marriage**	**Divorce**
**Education**			
	**Illiterate**	2 (0.1)	166 (2)
	**Less than diploma**	225 (6)	2635 (31.9)
	**Diploma**	724 (19.2)	2965 (35.8)
	**Associate degree**	300 (8)	552 (6.7)
	**Bachelor**	1695 (44.9)	1522 (18.4)
	**Master**	653 (17.3)	352 (4.3)
	**Ph.D.**	174 (4.6)	80 (1)
**Job**			
	**Unemployed**	899 (21)	398 (5)
	**Housewife**	772 (18)	3718 (47.1)
	**Self-employed**	1589 (37.1)	2334 (29.6)
	**Employee**	1023 (23.9)	1444 (18.3)
**Being a resident**			
	**Resident**	3466 (80.5)	7745 (93.2)
	**Non-resident**	842 (19.5)	563 (6.8)
Data presented as n (%)			

**Table 2:** Socioeconomic and demographic information of participants that selected for second phase


**Variables**		**Divorced**	**Married**	**P-value**
**Job**				
	**Unemployed**	42 (14)	61 (20.3)	
	**Housewife**	99 (33)	43 (14.3)	
	**Employee**	42 (14)	74 (24.7)	
	**Self-employed**	117 (39)	122 (40.7)	< 0.001
**Education**				
	**Less than diploma**	71 (23.6)	27 (9)		
	**Diploma**	95 (31.6)	67 (22.3)		
	**Academic**	134 (44.6)	206 (68.6)	< 0.001
**Age difference (yr)**				
	**≤ 5**	162 (54)	193 (64.3)		
	**5-10**	95 (31.6)	89 (29.8)		
	**> 10**	43 (14.3)	18 (6)	< 0.001
**Age difference (gender)**				
	**Man**	212 (71.7)	257 (88.3)		
	**Woman**	88 (28.3)	43 (11.7)	< 0.001
**Being a resident**				
	**Resident**	216 (71.8)	249 (83)	
	**Nonresident**	84 (28.2)	51 (17)	< 0.001
**Gender**				
	**Woman**	148 (49.3)	145 (48.3)	
	**Man**	152 (50.7)	155 (51.7)	0.87
Data presented as n (%). Chi-square test				

**Table 3:** The odds ratio of socio-economic factors that affecting the occurrence of divorce


		**Univariate model**		**Multivariable model**	
**Variables**		**OR (95% CI)**	**P-value**	**OR (95% CI)**	**P-value**
**Job**					
	**Unemployed**	Ref	1	Ref	1
	**Housewife**	3.344 (1.965-5.690)	< 0.001	1.505 (0.755-3.000)	0.24
	**Employee**	0.824 (0.478-1.423)	0.488	0.535 (0.267-1.070)	0.07
	**Self-employed**	1.393 (0.873-2.223)	0.165	0.488 (0.255-0.934)	0.03
**Education**					
	**Less than diploma**	Ref	1	Ref	1
	**Diploma**	0.399 (0.214-0.744)	0.004	0.626 (0.310-1.264)	0.19
	**Academic**	0.146 (0.082-0.260)	< 0.001	0.223 (0.116-0.430)	< 0.001
**Being a resident**					
	**Resident**	Ref	Ref	1
	**Nonresident**	1.916 (1.278-2.873)	0.002	2.163 (1.314-3.562)	< 0.001
**Age difference (yr)**					
	**≤ 5**	Ref	1	Ref	1
	**5-10**	1.253 (0.873-1.798)	0.222	1.362 (0.873-2.125)	0.17
	**> 10**	2.615 (1.436-4.762)	0.002	3.848 (1.803-8.213)	< 0.001
**Age difference (gender)**					
	**Man**	Ref	1	Ref	1
	**Woman**	2.984 (1.907-4.670)	< 0.001	3.403 (1.981-5.848)	< 0.001
Binary logistic regression model					

**Table 4:** The odds ratio of socio-economic factors that affecting the occurrence of divorce by gender


		**Woman**		**Man**	
**Variables**		**OR (95% CI)**	**P-value**	**OR (95% CI)**	**P-value**
**Job**					
	**Unemployed**	Ref	1	Ref	1
	**Housewife**	1.91 (0.86-4.25)	0.11	0.062 (0.006-0.62)	0.01
	**Employee**	0.19 (0.07-0.51)	0.001	1.94 (0.67-5.6)	0.22
	**Self-employed**	0.13 (0.05-0.35)	< 0.001	1.54 (0.58-4.1)	0.38
**Education**					
	**Less than diploma**	Ref	1	Ref	1
	**Diploma**	0.38 (0.12-1.24)	0.11	0.53 (0.19-1.42)	0.20
	**Academic**	0.23 (0.08-0.68)	0.008	0.11 (0.04-0.30)	< 0.001
**Being a resident**					
	**Resident**	Ref	Ref	1
	**Nonresident**	1.78 (0.84-3.75)	0.12	2.30 (1.14-4.62)	0.01
**Age difference (yr)**					
	**≤ 5**	Ref	1	Ref	1
	**5-10**	1.66 (0.83-3.32)	0.174	1.38 (0.73-2.60)	0.31
	**> 10**	2.78 (0.85-9.07)	0.08	3.15 (1.09-9.09)	0.03
Binary logistic regression, multivariate model					

## 4. Discussion

This study aimed to investigate the changes in marriage, childbearing, and divorce in Yazd province from April 2016 to March 2021 and determine the socioeconomic and demographic factors affecting the occurrence of divorce in this province using the available data. According to the findings of this article, the number of marriages and childbearing in Yazd province has decreased. Besides, this province had a growing increase in divorce cases. According to the analysis of individual factors in economic and social characteristics, the following factors played a decisive role in predicting the odds of divorce: education, occupation, being a resident, age difference, and the older of the wife compared to the husband.

The study found that there was a correlation between education level and the likelihood of divorce. Individuals who had less than a diploma, including those who were illiterate, only had an elementary, middle school, or high school education, were more likely to experience divorce compared to those who educated from a university. The odds of divorce decreased as the level of education increased. Other studies showed an inverted U-shaped relationship between education level and divorce, with individuals who had a diploma being 40% more likely to be divorced than university graduates. Moreover, several studies have demonstrated that couples with higher education levels are better equipped to respond appropriately to life situations and are more capable of solving problems (14, 19, 20).

In contrast to our study results, other researchers found that as education levels, especially for women, increased, so did the likelihood of divorce. This could be because higher education provides women with more knowledge about their rights, leading them to seek divorce if they find their lives unfavorable (15, 21).

The study also found that economic stability played a significant role in preventing divorce. Individuals who were employed or self-employed, or had non-government jobs, had a lower likelihood of experiencing divorce, with odds decreasing by 47% and 52%, respectively. On the other hand, being unemployed was associated with an increased risk of divorce. Other studies have also shown a positive correlation between unemployment and divorce rates (22, 23). Conversely, employment contributed to the stability and continuity of life together, as confirmed in other studies (24, 25).

The study found that being a housewife was associated with a higher incidence of divorce, although this association was not statistically significant. According to another study that has been done, housewives were more likely to get divorced than working women (26). This could be because housewives feel deprived of many social situations, which can lead to a sense of loss and increase their tendency to divorce. In summary, women who are employed can enjoy several advantages, including economic stability, a sense of self-worth and independence, shared household and childcare responsibilities, and social support. These benefits can enhance their overall well-being and contribute to the stability of their marriage.

Furthermore, it was discovered in our research that a difference in age exceeding 10 yr amplified the likelihood of divorce by 3.8 times. This discovery corroborated the findings of another study (27). However, contrasting these results, another study revealed that divorces were less frequent when there was an age difference exceeding 5 yr (28). The considerable age disparity within couples may result in a dearth of mutual comprehension, interaction, and alignment of their desires and requirements, thereby elevating the probability of divorce.

Previous studies have also reported that age disparity within a relationship can have a significant impact on divorce rates. The current research builds upon these findings by specifically exploring the role of gender in this dynamic. The patriarchal norms present in the society being examined appear to perpetuate the notion that older men are more desirable partners, leading to a power imbalance within relationships where the woman is older. This imbalance may contribute to increased dissatisfaction and instability within the marriage, ultimately increasing the likelihood of divorce (28).

Moreover, the study revealed that differences in the couple's residence were associated with a more than 2-fold increase in the odds of divorce. According to another research, 93.3% of divorce applicants cited cultural differences and a lack of understanding as the main reasons for their divorce (29). The findings demonstrate that a lack of mutual understanding regarding social, religious, cultural, and regional values can lead to tensions that may eventually result in divorce.

As stipulated by relevant organizations, preserving the confidentiality of individuals' information during marriage and divorce prevented us from incorporating additional variables that could have indicated socioeconomic status, cultural differences, psychological factors such as personality disorders, violence, and addiction. Furthermore, we did not address the family background, sociocultural status of couples, such as poverty, forced and pre-planned marriages, which predominantly affect girls under the age of 20 and can significantly contribute to marital breakdown. To better understand the causes of divorce differences, further qualitative and mixed-method studies are necessary.

## 5. Conclusion

The results of our study showed that, in the last 5 yr (2016-2021), marriage and childbearing had decreased, whereas the frequency of divorce increased in Yazd populations. Stability in job status and increasing the level of education could reduce the odds of divorce. Also, in non-native marriages, an age difference of more than 10 yr between couples and the woman being older than her husband can increase the odds of divorce. Therefore, adopting policies appropriate to Iranian society's economic, social, and cultural conditions, and the necessary planning in education and counseling before marriage is an inevitable necessity.

##  Conflict of Interest

The authors declare that there is no conflict of interest.
